# HLA-B*44 is associated with a more than twentyfold increase in cyclic citrullinated peptide antibody serum level in Croatian patients with seropositive rheumatoid arthritis

**DOI:** 10.1371/journal.pone.0334551

**Published:** 2025-12-01

**Authors:** Jure Aljinović, Darko Kero, Daniela Šošo, Marin Petrić, Daniela Marasović Krstulović, Dijana Perković, Esma Čečuk-Jeličić, Sonja Jaman, Dora Dujmović, Ivanka Marinović

**Affiliations:** 1 Division of Physical Medicine and Rehabilitation with Rheumatology, University Hospital of Split, Split, Croatia; 2 University of Split School of Medicine, Split, Croatia; 3 Faculty of Health Sciences, University of Split, Split, Croatia; 4 Study Program of Dental Medicine, University of Split School of Medicine, Croatia; 5 Department of Internal Medicine, Division of Rheumatology, Allergology and Clinical Immunology, University Hospital of Split, Croatia; 6 Division of Transfusion Medicine, University Hospital of Split, Croatia; Kyoto University Graduate school, JAPAN

## Abstract

**Objectives:**

To determine whether any of the HLA-A*, HLA-B* and HLA-DR* alleles in seropositive rheumatoid arthritis (RA) can be associated with the extreme serum levels of rheumatoid factor (RF) and cyclic citrullinated peptide antibodies (anti-CCP).

**Methods:**

This was a retrospective cross-sectional study of adult patients. Demographic data, HLA typing data and RF and anti-CCP levels were collected and analysed.

**Results:**

HLA-A*02, HLA-B*44 and HLA-DRB1*04 were more prevalent in patients from the RA cohort compared to healthy controls. Intra-cohort analysis revealed that HLA-B*44 had an increased frequency of a twenty-fold increase in anti-CCP (P = 0.033) and HLA-A*03 had a borderline (P = 0.063) frequency. HLA-A*03 was more frequent in the groups with >3-fold and >10-fold anti-CCP levels, while HLA-DRB1*04 was of borderline significance at >3-fold anti-CCP increase (P = 0.053). HLA-B*08 showed a lower frequency of 3-, 10- and 20-fold increase in anti-CCP serum levels. Carriers of B*44 (OR = 5.58; P = 0.030), DRB1*04 (OR = 3.89; P = 0.027) or A*03 (OR = 2.71; P = 0.023) were statistically significantly more likely to have a 20-fold increase in anti-CCP levels over the diagnostic threshold. None of the alleles increased the likelihood of having high or extreme RF levels.

**Conclusions:**

HLA-B*44 is associated with a twenty-fold increase in anti-CCP serum levels. HLA-A*03 and HLA-DRB1*04 have a positive trend towards a twenty-fold increase in anti-CCP levels. Earlier aggressive therapy in these patients can prevent such an extreme increase in antibody levels.

## Introduction

The DRB1*04 allele of the human leukocyte antigen (HLA) system is a known risk factor for the development of rheumatoid arthritis (RA) [1] and is associated with increased serum levels of antibodies against cyclic citrullinated antibodies (anti-CCP) together with HLA-DRB1 *01 and HLA-DRB1*10 alleles. The suspected mechanism is the „shared epitope (SE)“, a 5-amino acid motif on the β1 chain of the HLA-DR molecules. Genes for SE are found in these alleles: HLA-DRB1*04:01,*04:04, *04:05, (HLA-DR4), HLA-DRB1*01:01 (HLA-DR1), HLA-DRB1*10:01 (HLA-DR10) [1], and are present in about two-thirds of seropositive RA patients [2].

Epitopes of HLA-DRB1*0101 and *0401 have been associated with more aggressive disease progression in RA, both globally and in Croatia [3,4]. Recently, some of the non-SE alleles (HLA-DRB1*09 and HLA-DRB1*15) have been associated with higher levels of anti-CCP compared to the other non-SE alleles [5].

Previously, our team had reported that HLA-B*44-positive patients referred to a rheumatology outpatient clinic are more likely to develop peripheral joint disease (about 50%) [6]. HLA-B*44 is a very common HLA-B loci allele worldwide, the third most common in Croatia with an incidence of about 10% and is not specific for any disease [7,8]. However, its presence has been shown to modulate the course of infectious and autoimmune diseases (neurological, rheumatological and immunological diseases) [9–15]. When analysing subgroups in our B44 cohort, 6.9% of all patients had seropositive RA (n = 21) [6]. Most of the anti–CCP and rheumatoid factor (RF) values were above the maximum level that could be detected in our laboratory. For this reason, we decided to compare the values of anti-CCP and RF in patients with seropositive RA in Split-Dalmatia County, considering HLA B*44 positivity and other HLA-B alleles.

The limitation of all quantitative studies is that it is not possible to accurately measure values below and above the diagnostic threshold and in these cases the values are reported as negative or greater than the maximum measurable value. In addition, the different tests give different reference values and are therefore not comparable. Therefore, a comparison of the number of exceedances of the diagnostic threshold of positivity seems to be more meaningful. Even the ACR/EULAR classification criteria for RA assess the presence of RF and anti-CCP antibodies as negative, low positive (1–3-fold increase) and high positive (3 or more times the threshold) and do not include absolute values [16].

On the other hand, we believe that clinically significant data can be found if the anti-CCP and RF values are categorised into several groups. Therefore, we formed five groups: negative, 1–3-fold, 3–10-fold, 10–20-fold and more than 20-fold increase.

In this study, we aimed to determine whether the presence of the HLA-B*44 allele in seropositive RA patients could be associated with the higher serum levels of RF and anti-CCP. As a secondary aim, we aimed to identify other HLA-B*, HLA-A* and HLA-DRB1* alleles in seropositive RA patients that could also be associated with the higher serum levels of RF and anti-CCP. The identification of new HLA alleles as predictors of high serum levels of RF and anti-CCP is necessary for future stratification of RA patients with respect to antibody levels and prognosis of RA progression. Our hypothesis is that besides HLA-DRB1*, other HLA-A* and HLA-B* alleles, especially HLA-B*44, could be novel independent predictors contributing to the strong increase of RF and anti-CCP serum levels in RA patients.

## Materials and methods

### Study participants

A retrospective cross-sectional analysis of data from RA-positive patients was performed on adult patients of both genders, recruited from the Department of Rheumatology and Clinical Immunology and the Department of Physical and Rehabilitation Medicine with Rheumatology at the Split Clinical Hospital Centre in Croatia between 1 May 2018 and 30 January 2025. The study was conducted in accordance with the Declaration of Helsinki and approved by the Ethics Committee of the University Hospital Centre Split (520–03/24–01/25, 22.02.2024. and 520–03/24–01/244, 31.12.2024.). The data were accessed from the medical records in the time period from the 01.01.2025–01.04.2025 by the principal investigator. After that, the patients were coded and data was analysed by other co-authors. Patient consent was waived by the Ethical Committee with the obligation that all patient data were fully anonymized, and patients were coded with identities known only to the principal investigator. The dataset is available as S1 Dataset.

The inclusion criterion for the study was seropositive RA diagnosed according to the EULAR/ACR 2010 criteria and containing data on the HLA A*, HLA B* and HLA DR* alleles [16]. Exclusion criteria were seronegative RA and patients with isolated RF or anti-CCP increase who did not fulfil the EULAR/ACR 2010 criteria. The data on the use of a biological therapy were collected as a surrogate measure for a more severe disease (biological drug – more severe disease).

### HLA typing

Extraction of genomic DNA from EDTA blood samples was performed using the High Pure PCR Template Preparation Kit (Roche Diagnostics GmbH, Germany) at the Department of Transfusion Medicine, University Hospital Centre Split, Croatia. Either PCR sequence-specific oligonucleotide probe hybridisation (PCR-SSO) or standard polymerase chain reaction sequence-specific priming (PCR-SSP) methods were used to determine HLA-B and HLA-DRB1 alleles. PCR-SSO was performed using commercially available typing kits (Immucor Transplant Diagnostics, Inc., Stamford, USA) on the Luminex Multi-Analyte Profiling System (xMAP, Luminex Corporation, TX, USA), and HLA typing was performed using MATCH IT!® DNA software (Immucor Transplant Diagnostics, Inc., Stamford, USA) provided by the manufacturer. Genotyping with the PCR-SSP method was performed using the standard protocol for the Olerup SSP® typing kits (Olerup GmbH, Vienna, Austria) and the results were analysed using Helmberg-SCORE 5 software (CareDx Inc., Brisbane California, USA).

### RF and anti-CCP serum levels measurements

Plasma concentration of RF was measured using a standard laboratory method on the Roche Cobas 8000 (≥14 IU/mL positivity cut-off), and anti-CCP was measured by chemiluminescence on the Abbott Alinity ci-Series analyser (≥5.0 U/mL positivity cut-off, lowest measurable value <0.5 U/mL, highest >195 U/mL).

The RF serum level groups were formed according to how often the RF value exceeds the positivity cut-off value: 0: normal value; 1: from 14–42 IU/ml (1 –3×); 2: 43–140 IU/ml (3 –10×); 3: 140–280 IU/ml (10 –20×) 4. > 281 IU/ml (> 20×).

The groups for the anti-CCP serum level were formed according to the number of times the cut-off value for positivity was exceeded: 0: normal value; 1: 5.0 to 15.0 U/ml (1 –3×); 2: 16.0–50 U/ml (3 –10×); 3: 51–102 IU/ml (10 –20×); > 103 IU/ml (>20×).

### Statistical analysis

The demographic parameters and the frequency of HLA alleles in the RA cohort were analysed using descriptive statistics. The comparison of the frequency of HLA alleles between the RA cohort and the healthy bone marrow donors from the Croatian national database was performed using the Chi-squared test. Comparison between the frequency of biologic drug usage between HLA alleles and RA cohort was performed using the Chi-squared test.

The differences in RF and anti-CCP values between B*44-positive RA patients and the rest of the RA cohort were analysed using simple linear regression, with RF and anti-CCP as outcome variables and a single predictor transformed into a dichotomised „dummy“ variable, for which membership of the B*44 subgroup was coded as “1“ and the rest of the cohort as “0“. For this analysis, the values of RF and anti-CCP were logarithmically transformed to normalise their distribution.

The association between the different HLA alleles (predictors) and the changes in RF and anti-CCP scores as outcome variables was analysed using multinomial logistic regression, where RF and anti-CCP scores were transformed into nominal outcome variables with five different categories: normal values (reference variable), 1–3 × , 3–10 × , 10–20× and >20 × increase.

The statistical significance for all tests was set at α = 0.05 (P < 0.05). Even when multiple tests were necessary, *P-*values were reported as nominal values, and assessed using the Benjamini-Hochberg correction with the False Discovery Rate (FDR) set at 0.05, and the significance was defined as q-value < FDR. The statistical analysis was performed in Microsoft Excel 2016 (Microsoft Corporation, Redmond, WA, USA).

## Results

A total of 174 patients with seropositive RA were included in this study, with a mean age of 59.74 ± 11.21 (SD). The RA cohort consisted of 80.46% (140) women and 19.54% (34) men.

**Fig 1:**
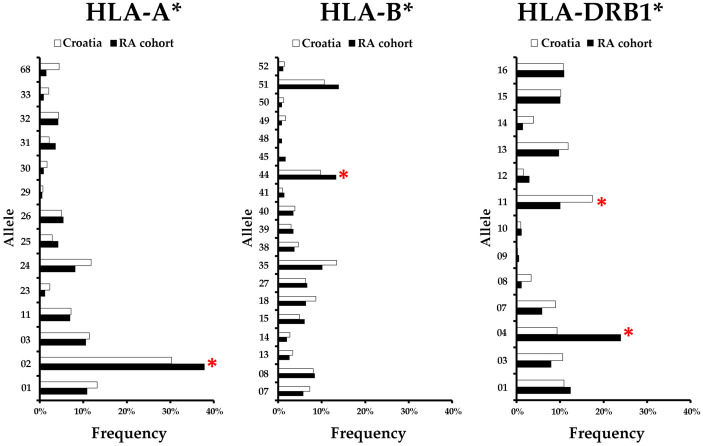
Frequency of HLA-A*, HLA-B* and HLA-DRB1* alleles in the RA cohort and in the Croatian population of bone marrow donors. Red asterisks indicate a statistically significant difference between the frequency of the allele in the cohort and in the population.

Compared to the Croatian population of bone marrow donors, alleles HLA-A*02, HLA-B*44 and HLA-DRB1*04 were more frequent in the RA cohort, while HLA-DRB1*11 was less frequent in the RA cohort (Fig 1). The data of all HLA-A, B and DR frequencies that were present in more than 20 cases in the RA group are shown in Table 1.

**Table 1:** Comparison of frequencies of a subset of HLA-A*, HLA-B* and HLA-DRB1* alleles between patients with rheumatoid arthritis and the Croatian population of bone marrow donors.

Alleles		RA cohort		Croatia^†^		χ^2^	*P*-value^‡^
Group	**Type**	n	%	n	%		
HLA-A*	01	36	10.91	1318	13.18	1.13	0.287
	**02**	125	37.88	3026	30.26	4.40	**0.036**
	03	35	10.61	1148	11.48	1.69	0.193
HLA-B*	08	29	8.43	806	8.06	0.05	0.819
	27	23	6.69	630	6.30	0.07	0.786
	35	35	10.17	1342	13.42	2.39	0.122
	**44**	46	13.37	973	9.73	3.95	**0.047**
	51	48	13.95	1062	10.62	2.07	0.150
HLA DRB1*	01	42	12.43	1092	10.92	0.60	0.438
	**04**	81	23.96	934	9.34	23.93	**<0.001**
	**11**	34	10.06	1742	17.42	9.55	**0.002**
	15	34	10.06	1019	10.19	0.01	0.944
	16	37	10.95	1074	10.74	0.01	0.913

*Alleles present in more than 20 patients from the RA cohort; ‡ Chi-squared test (α = 0.05; P < 0.05; df = 1), † Croatian population of 10 000 bone marrow donors.

The possible association between HLA-A*, HLA-B* and HLA-DRB1* alleles present in more than 20 patients and the increase in RF (Table 2) and anti-CCP levels (Table 3) was investigated. The increase in RF levels for most alleles was comparable to the average of the RA cohort, apart from HLA-A*03. In patients with the HLA-A*03 allele, RF levels were increased three-fold or more compared to the rest of the RA cohort. In contrast, 90% of patients with the HLA-B*08 allele had up to a 10-fold increase in RF levels (χ2 = 8.81; P = 0.003), making them less frequent in the group with more than a 10-fold increase. For the HLA-DRB1* alleles, all alleles showed RF levels comparable to the RA cohort.

**Table 2:** Breakdown of RF levels in the RA cohort according to different HLA alleles.

Alleles	RF increase groups*					Patients (n)
	Normal (n)	1-3× (n)	3-10× (n)	10-20× (n)	>20× (n)	
RA cohort	4.1% (7)	28.1% (48)	29.2% (50)	20.5% (35)	18.1% (31)	171
B*44	4.8% (2)	19.0% (8)	33.3% (14)	23.8% (10)	19.0% (8)	42
B*51	4.8% (2)	35.7% (15)	11.9% (5)	23.8% (10)	23.8% (10)	42
B*35	3.3% (1)	26.7% (8)	23.3% (7)	26.7% (8)	20.0% (6)	30
B*27	0.0% (0)	17.4% (4)	43.5% (10)	26.1% (6)	13.0% (3)	23
B*08^†^	3.4% (1)	41.4% (12)	44.8% (13)	**3.4% (1)**	**6.9% (2)**	29
DRB1*04	1.4% (1)	31.0% (22)	19.7% (14)	25.4% (18)	22.5% (16)	71
DRB1*01	0.0% (0)	25.0% (10)	32.5% (13)	25.0% (10)	17.5% (7)	40
DRB1*16	8.3% (3)	27.8% (10)	19.4% (7)	19.4% (7)	25.0% (9)	36
DRB1*15	3.3% (1)	20.0% (6)	43.3% (13)	16.7% (5)	16.7% (5)	30
DRB1*11	3.0% (1)	30.3% (10)	24.2% (8)	21.2% (7)	21.2% (7)	33
A*02	6.3% (6)	31.6% (30)	28.4% (27)	15.8% (15)	17.9% (17)	95
A*01	9.1% (3)	36.4% (12)	33.3% (11)	12.1% (4)	9.1% (3)	33
A*03^†^	**0.0% (0)**	**13.8% (4)**	41.4% (12)	17.2% (5)	27.6% (8)	29

*1–3 × : 14–42 IU/ml; 3–10 × : 43–140 IU/ml; 10–20 × : 140–280 IU/ml; > 20 × : > 281 IU/ml. Frequencies with statistical differences (Chi-squared test, α = 0.05; *P* < 0.05) are shown in bold. Multiple testing across the five titer groups between the entire RA cohort and patient groups with the 13 alleles were controlled with the Benjamini-Hochberg correction procedure (FDR = 0.05; m = 13). † Alleles where only the top-ranked comparison among the 13 tests meets the Benjamini-Hochberg criterion (*q* < FDR).‡ Comparisons that remain significant after Benjamini-Hochberg correction (*q* < FDR).

**Table 3:** Breakdown of anti-CCP levels in the RA cohort according to different HLA alleles.

Alleles	Anti-CCP increase groups*					Patients (n)
	Normal (n)	1-3× (n)	3-10× (n)	10-20× (n)	>20× (n)	
RA cohort	14.4% (25)	5.7% (10)	14.9% (26)	13.2% (23)	51.7% (90)	174
B*44	7.0% (3)	4.7% (2)	9.3% (5)	9.3% (4)	**69.8% (29)**	43
B*51	9.5% (4)	7.1% (3)	23.8% (10)	9.5% (4)	50.0% (21)	42
B*35	6.7% (2)	3.3% (1)	16.7% (5)	16.7% (5)	56.7% (17)	30
B*27	17.4% (4)	4.3% (1)	4.3% (1)	17.4% (4)	56.5% (13)	23
B*08^†^	34.5% (10)	6.9% (2)	20.7% (6)	6.9% (2)	**31.0% (9)**	29
DRB1*04	7.0% (5)	2.8% (2)	18.3% (13)	14.1% (10)	57.7% (41)	71
DRB1*01	12.5% (5)	5.0% (2)	10.0% (4)	12.5% (5)	60.0% (24)	40
DRB1*16	2.8% (1)	11.1% (4)	16.7% (6)	13.9% (5)	55.6% (20)	36
DRB1*15	12.9% (4)	0.0% (0)	16.1% (5)	12.9% (4)	58.1% (18)	31
DRB1*11	18.2% (6)	3.0% (1)	15.2% (5)	6.1% (2)	57.6% (19)	33
A*02	14.4% (14)	7.2% (7)	16.5% (16)	14.4% (14)	47.4% (46)	97
A*01	24.2% (8)	6.1% (2)	15.2% (5)	9.1% (3)	45.4% (15)	33
A*03	**0.0% (0)**	**0.0% (0)**	16.7% (5)	13.3% (4)	70.0% (21)	30

*1–3 × : 5.0–15.0 U/ml; 3–10 × : 16.0–50 U/ml; 10–20 × : 51–102 IU/ml; > 20 × : > 103 IU/ml. Frequencies with statistical differences (Chi-squared test, α = 0.05; *P* < 0.05) are shown in bold. Multiple testing across the five increase-groups between the entire RA cohort and patient groups with the 13 alleles were controlled with the Benjamini-Hochberg correction procedure (FDR = 0.05; m = 13). † Alleles where only the top-ranked comparison among the 13 tests meets the Benjamini-Hochberg criterion (*q* < FDR). ‡ Comparisons that remain significant after Benjamini-Hochberg correction (*q* < 0.05).

The 20-fold increase in anti-CCP levels was present in 51.7% of patients from the cohort with end-stage RA. According to the within-cohort analysis of patients with elevated anti-CCP levels, the 20-fold increase in anti-CCP levels was most frequently observed in patients with HLA-B*44 and HLA-A*03 alleles (7 out of 10 patients). The frequency of patients with a 20-fold increase in anti-CCP levels was statistically significantonly for the HLA-B*44 allele (χ2 = 4.55; P = 0.033), while it was borderline for the HLA-A*03 allele (χ2 = 3.46; P = 0.063). However, after Benjamini-Hochberg correction at FDR = 0.05, this comparison did not remain significant (q ≥ 0.05). For other analysed titres, patients with the HLA-A*03 allele were more likely than others to have a 3- to 10-fold or higher increase in anti-CCP levels (χ2 = 7.27; P = 0.007 and χ2 = 3.95; P = 0.047, respectively), but also without significance after Benjamini-Hochberg correction. In contrast, the 20-fold increase in anti-CCP levels in patients with other HLA-A* and HLA-B* alleles were comparable to the average of the RA cohort.

It should be noted that patients with the HLA-B*08 allele had a lower-than-average frequency of more than 3-fold (χ2 = 6.31; P = 0.012), 10-fold (χ2 = 7.55; P = 0.006) and 20-fold (χ2 = 4.26; P = 0.039) increase in anti-CCP. This suggests that HLA-B*08 may play a protective role by maintaining low RF and anti-CCP levels above the diagnostic threshold. Looking at the HLA-DRB1* alleles, patients with different HLA-DRB1* alleles showed an increase in anti-CCP levels that was mostly comparable to the average of the RA cohort, with the exception of patients with the HLA-DRB1*04 allele, who showed a more than 3-fold increase in anti-CCP levels above the average of the RA cohort, albeit with borderline statistical significance (χ2 = 3.74; P = 0.053).

**Fig 2:**
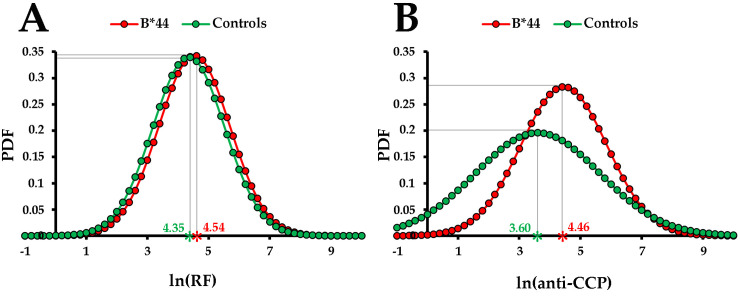
Distributions of RF (A) and anti-CCP (B) levels in patients with rheumatoid arthritis with HLA-B*44 and other HLA-B* alleles (controls). The levels for RF and anti-CCP were logarithmically transformed (natural logarithm). The asterisks indicate the mean logarithmic levels of RF and anti-CCP in patients with HLA-B*44 (red) and other HLA-B* alleles (green) (x-axis); (PDF – Probability Density Function) (y-axis).

As patients with the HLA-B*44 allele were more likely to have a 20-fold increase in anti-CCP levels compared to the rest of the RA cohort (other HLA-B* alleles), we further investigated whether there was an overall correlation between the HLA-B*44 allele and increased anti-CCP levels by simple linear regression (Fig 2, Table 4). On average, patients from the entire cohort had relatively high RF and anti-CCP levels. However, patients with the HLA-B*44 allele had statistically significantly higher anti-CCP levels than the rest of the RA cohort. Thus, the presence of the HLA-B*44 allele in patients with RA was on average correlated with more extreme anti-CCP levels, but not with extreme RF levels.

**Table 4:** Comparison of RF and anti-CCP levels in patients with rheumatoid arthritis with B*44 and other HLA B* alleles (controls).

OUTCOME	„Dummy“variables (categories)	PREDICTORS		Model parameters	
		Coefficients*	95% CI	R	*P*-value^†^
RF^‡^	Controls	4.35	4.14; 4.56	0.01	0.387
	B*44	+0.19	−0.24; 0.61		
anti-CCP^§^	Controls	3.60	3.26; 3.95	0.19	**0.015 (< 0.05)**
	B*44	**+0.86**	0.17; 1.54		

*Mean values of outcomes for the reference category (controls) and mean deviation from these values (positive/negative) in patients with rheumatoid arthritis with genotype B*44; †Simple linear regression (α = 0.05; P < 0.05; df = 1); ‡Outcome – logarithmically transformed RF values for both HLA-B*44 patients and controls (n = 160); §Outcome – logarithmically transformed anti-CCP values for both HLA-B*44 patients and controls (n = 160).

Finally, the association between the HLA-A*, HLA-B* and HLA-DRB1* alleles that were statistically significantly (or borderline) more frequent in patients from the RA cohort with extreme anti-CCP levels was further analysed using multinomial logistic regression (Table 5). According to this analysis, patients from the RA cohort with either B*44 (OR = 5.58; P = 0.030), DRB1*04 (OR = 3.89; P = 0.027) or A*03 (OR = 2.71; P = 0.023) alleles were statistically significantly more likely to have more than 20 times the normal anti-CCP levels. Consistent with the results shown previously, the presence of B*44, DRB1*04 or A*03 did not statistically significantly increase the odds of having high or extreme RF levels in patients from the RA cohort.

**Table 5:** Association between HLA-B*44, HLA-DRB1*04 and HLA-A*03 alleles and different RF and anti-CCP levels in patients with seropositive rheumatoid arthritis (multinomial logistic regression).

OUTCOMES*	PREDICTORS^†^	Coefficients	OR^‡^	95% CI		*P*-value^§^
				Lower	Upper	
RF (14–42 IU/ml) 1-3×	B*44	−0.96	0.38	0.06	2.45	0.309
	DRB1*04	1.67	5.30	0.58	48.22	0.583
	A*03	−1.12	0.32	0.11	1.01	0.050
RF (43–140 IU/ml) 3-10×	B*44	−0.07	0.93	0.16	5.56	0.939
	DRB1*04	0.84	2.31	0.25	21.39	0.462
	A*03	0.62	1.86	0.81	4.28	0.143
RF (140–280 IU/ml) 10-20×	B*44	0.01	1.01	0.16	6.39	0.994
	DRB1*04	2.00	7.40	0.78	69.84	0.080
	A*03	−0.17	0.84	0.29	2.42	0.748
RF (>281 IU/ml) >20×	B*44	−0.06	0.94	0.14	6.19	0.946
	DRB1*04	1.76	5.79	0.59	56.11	0.129
	A*03	0.81	2.25	0.87	5.79	0.095
anti-CCP (5.-15 IU/ml) 1-3×	B*44	0.93	2.55	0.30	21.39	0.388
	DRB1*04	0.68	1.98	0.35	11.23	0.439
	A*03	0.97	2.63	0.31	21.92	0.373
anti-CCP (16–50 IU/ml) 3-10×	B*44	0.94	2.55	0.43	15.21	0.305
	DRB1*04	1.71	**5.52**	1.43	21.32	**0.013**
	A*03	0.15	1.16	3.39	15.11	0.791
anti-CCP (51–102 IU/ml) 10-20×	B*44	0.79	2.19	0.35	13.69	0.400
	DRB1* 04	1.36	3.89	0.99	15.31	0.052
	A*03	−0.06	0.94	0.29	3.01	0.921
anti-CCP (>103 IU/ml) >20×	B*44	1.72	**5.58**	1.18	26.51	**0.030**
	DRB1*04	1.36	**3.89**	1.17	12.98	**0.027**
	A*03	0.99	**2.71**	1.15	6.40	**0.023**

* RF and anti-CCP values divided into four categories with reference categories referring to the normal RF and anti-CCP levels; †Predictors B*44, DRB1*04 and A*03 were defined as dichotomous variables (0 = no allele; 1 = allele present); ‡Odds Ratio; §Multinomial logistic regression (α = 0.05; P < 0.05; df = 3) (n = 160).

In 43.68% of seropositive RA patients, a biological drug was prescribed. The use of biologic therapy was analysed as a surrogate measure of higher disease activity. There were no statistically significant differences between the most common alleles and the percentage of biologic therapy use (Table 6).

**Table 6:** Use of biological therapy according to different HLA alleles in RA cohort patients.

HLA Allele	Therapy			χ^2^	*P-value* ^†^	*q* ^‡^
	Biological (n)	Total (n)	Biological (%)			
A*01	20	33	60.61%	1.09	0.297	0.933
A*03	12	30	40.00%	0.06	0.811	
A*02	39	97	40.21%	0.13	0.723	
B*51	15	42	35.71%	0.37	0.542	
B*35	12	30	40.00%	0.05	0.811	
B*27	5	23	21.74%	1.92	0.166	
B*08	15	29	51.72%	0.24	0.625	
B*44	15	43	34.88%	0.46	0.495	
DRB1*04	32	71	45.07%	0.01	0.901	
DRB1*01	17	40	42.50%	0.01	0.932	
DRB1*16	15	36	41.67%	0.02	0.889	
DRB1*15	14	31	45.16%	0.01	0.924	
DRB1*11	14	33	42.42%	0.01	0.933	
RA cohort	76	174	43.68%	/	/	/

^†^Chi-squared test (α = 0.05; P < 0.05; df = 1). The significance of *P*-values were adjusted for multiple testing across all alleles (m = 13) assessed using the Benjamini-Hochberg correction (FDR = 0.05). ‡ Benjamini-Hochberg adjusted *q*-values (*q*) are reported in the rightmost column. No comparison met *q* < 0.05 (FDR).

## Discussion

Based on previous findings that the level of anti-CCP antibodies is relatively stable during the first five years of RA disease [17], in this work we aimed to find out whether there are HLA class I genes that induce higher anti-CCP levels in RA patients, regardless of whether the patient is a carrier of SE alleles or not. Our results showed that the presence of HLA-B*44 increases anti-CCP serum levels in 70% of patients by more than twenty-fold over the cut-off value, compared to 40–60% for all other HLA-B genes. We have shown that this substantial increase in anti-CCP levels cannot be attributed to the SE alleles and that some of the class I HLA antigens should also be considered, in particular the HLA-B*44 allele. The presence of the HLA-B*44 allele had an odds ratio of 5.58 for a 20-fold increase in anti-CCP. Surprisingly, HLA-A*03 showed a similar 70% increase with a 20-fold increase in anti-CCP levels, but with borderline statistical significance. For RF serum levels, we found no differences between the HLA-A, -B or DR alleles, except for the protective effect of HLA-B*08 on a more than 10-fold increase in anti-CCP.

SE alleles are known to increase the risk of developing RA in a study of identical twins, with the risk being even greater if the individual is homozygous for SE [18]. SE alleles are present in about 80% of anti-CCP-positive RA patients, while their frequency in anti-CCP-negative RA patients has lower to about 50–70% [19]. In our study, two thirds of all patients were HLA-DRB*01, DRB1*04 or DRB1*10 positive, with DRB1*04 being the most common with a frequency of 25%. This is in contrast to the study previously published in Croatia, which describes DRB1*0101 as the most frequent allele with 43% in RA patients [20], but in line with the meta-analysis by Stahl et al. from 2010 regarding the Caucasian population [21]. It has been described that SE alleles are associated with higher absolute anti-citrullinated protein antibodies (ACPA) levels [22]. However, some studies have shown that not only SE epitope alleles such as DRB1 *01, *04 and *10, but also non-SE alleles such as DRB1 *09, *15 were associated with higher absolute levels of ACPA antibodies [5]. Although in our study the HLA-DRB1 alleles did not differ from the cohort average of the allocation group for both anti-CCP and RF levels, the presence of HLA-DRB1*04 showed an odds ratio of 5.52 for the 3–10-fold increase group and 3.89 for the more than 20-fold increase group.

The current hypothesis is that SE sequences enable the presentation of self-antigens to B- or T–lymphocytes [1], more specifically citrullinated peptides [23]. So far, it has not been proven that citrullinated peptides bind better to SE alleles than to non-RA-associated HLA-DR alleles [24].

Some groups are investigating Peptydil Arginyl Deiminase 4 (PAD4) complex, that carries out post-translational modifications of proteins [25–28]. PAD4 converts arginine to citrulline in some proteins in people prone to RA. PAD4 antibodies are detected in 25–45% of established RA patients [25–28]. The latest hypothesis states that citrullinated proteins are only presented to antigen-presenting B lymphocytes if they are present in complex with PAD4. This is the hapten-carrier model, in which citrullinated peptides (which serve as haptens) are incorporated into the PAD4 complex (which serves as a carrier). They are then recognised by the B-lymphocytes and the disease begins [2,24]. The definition of hapten is that it is an antigen that is not immunogenic by itself, but only in complex with other antigens that are carriers. According to this definition, antigen-presenting cells form a complex between DR alleles with citrullinated antigens and T-cell receptor (TCR). To activate CD4 + lymphocytes, additional co-stimulatory binding between CD80/86 and CD20 is required. In this way, helper CD4 + T-lymphocytes are activated and stimulate B cells into an activated form called plasmocytes, that produce antibodies directly against citrullinated peptides. Abatacept targets this co-stimulatory mechanism and is proposed as the most effective drug for some alleles such as HLA-DRB1 04:05 [29]. It could be the drug of choice for HLA-B*44 carriers who, we can argue, overstimulate the B-lymphocytes so that they produce extreme levels of anti-CCP, as observed in this study.

In the previous paragraph, the association between HLA class II alleles and seropositive RA was described, but HLA class I alleles and their effect on RA have recently received more attention. So far, only a single amino acid polymorphism in HLA B*08-Asp9 has shown a twofold increase in the development of ACPA+ RA [30]. HLA class I alleles are responsible for the immune response of cytotoxic CD8 + lymphocytes, which cause their proliferation and clonal expansion and ultimately find and kill the target cells through chemokine receptors and cytotoxic mediators. Cytotoxic T cells targeting citrullinated antigens were increased in ACPA + patients compared to healthy controls and ACPA– RA [31]. These CD8 + cells expressed activation markers such as CD69 and GPR56 and reduced the expression of PD-1 and TIM3 as inhibitory markers [31], and there were more clonally expanded CD8 + T-lymphocytes than in healthy controls. Under experimental conditions, antibody-mediated blocking of HLA class I molecules on CD8 + T-lymphocytes from the blood of RA ACPA+ patients resulted in non-activation of these cells when exposed to citrullinated peptides [31]. It is known that the synovium of RA+ patients has an increased number of CD8 + T-lymphocytes [32], with a subpopulation of memory T-cells that are activated on contact with citrullinated peptides [33]. The “vicious circle” can be triggered by activated cytotoxic lymphocytes that kill the cell, possibly with molecules such as perforin, and then citrullinated histones and parts of the DNA can persist extracellularly in neutrophil extracellular traps (NET) [34,35]. Memory T-cells, which are constantly stimulated by self‐antigens (citrullinated peptides), can contribute to the chronic inflammation in RA [31,33]. Our team tried to reverse this finding to find out which HLA-B allele contributes most to the increase in anti-CCP levels in RA patients. Of all the HLA class I alleles, the HLA-B*44 allele was the most frequent in seropositive RA patients, and two out of three patients also had the highest levels of anti-CCP that can be measured in our laboratory. With regard to our results, further studies are needed in the SE + HLA-B*44 allele to see if there are more activated autoreactive CD8 + T-lymphocytes than in other HLA class I alleles. It has been shown that SE is not the reason for such an increase in anti-CCP levels. Patients with SE and HLA-B*44 would probably benefit most from early and more aggressive therapy of RA, at least methotrexate, to reduce the production of proinflammatory cytokines and to prevent the development of autoimmune memory cells [36].

Apart from the SE alleles and HLA-B*44, a twenty-fold increase in anti-CCP levels was observed with the HLA-A*03 allele. It is widespread in Europe and was described many years ago as a secondary risk factor for myasthenia gravis [37]. HLA-A03 allele is associated with haemochromatosis, but has no influence on disease progression [38] and increased clearance of the hepatitis C virus [39]. We have not found any recently described relationships with systemic inflammatory rheumatic diseases such as RA. Patients carrying HLA-A*03 had both a 3-fold or more and a 10-fold or more frequent anti-CCP increase present than the cohort, but with borderline results for the 20-fold elevation. As the statistical significance was borderline, a larger number of patients is needed to assess significance, but it should not be discarded as a factor for the extreme increase of anti-CCP serum levels.

### Limitations of the study

In this study, we only had the data from the basic routine HLA typing available. Further subtyping of the DRB1*01, DRB1*04 and DRB1*10 alleles is necessary as not all subtypes occur in this SE family. The correlation of age at disease onset, disease stage, smoking, periodontitis and degree of joint damage should be considered in the following studies, as RF and CCP values alone do not provide sufficient insight into disease progression or severity. The reason for not including this data in this study was the lack of a registry for RA patients in Croatia and the unavailability of data prior to 2018 in online medical documentation. In many long-term patients, HLA-DRB1 alleles are missing, and only HLA-B typing is available. Full HLA typing was not a routine procedure for RA patients in Croatia, but sometimes we had ordered the data for the HLA-B locus to exclude the peripheral type of HLA-B27 + spondyloarthritis. This was particularly frequent in patients who were anti-CCP and RF negative at the onset of the disease when the diagnostic procedures were ordered. The number of 43 RA patients in the HLA-B*44 group was sufficient to statistically demonstrate that there was a difference in anti-CCP levels. However, this number is too small and could only apply to the Croatian population. Analysis of registries or other populations could show different results and is therefore welcome to confirm or correct our data. Until we increase the sample size or reproduce the same findings in an independent dataset our results refer to the Croatian population only. This is necessary to expand knowledge in this area of research and prepare rheumatologists for a more personalised approach to each patient. There was also a positive trend of a twenty-fold increase in anti-CCP levels in HLA-A*03, but only 30 patients had this allele, so a larger cohort is required for more accurate statistical data.

## Conclusions

This comprehensive study on HLA typing of seropositive RA patients established the association between HLA-B*44, HLA-A*03, HLA-DRB1*04 and extremely elevated anti-CCP serum levels. Of all HLA type I alleles, only HLA-B*44 significantly increased the frequency of a twenty-fold increase in anti-CCP levels, while HLA-A*03 and HLA-DRB1*04 were less significant. Carriers of HLA-B*44, HLA-DRB1*04 and HLA-A*03 had odds ratios of 5.58, 3.89 and 2.71 respectively for a 20-fold increase in anti-CCP levels. In these patients, early aggressive therapy should be recommended to prevent an extreme increase in antibody levels by inhibiting the development of autoimmune memory cells. The creation of a registry for patients with rheumatoid arthritis in Croatia could help us to establish additional links between disease severity, environmental factors and the extreme increase in anti-CCP and RF serum levels for different HLA alleles.

No significant correlations were found between RF levels and HLA A, B and DRB1.

## Supporting information

S1 Dataset Anonymized participant background information, HLA typing, anti-CCP, RF levels and biologic therapy usage. (XLSX)
